# Nomogram for Quantitatively Estimating the Risk of Fibrosis Progression in Type 2 Diabetic Patients With Nonalcoholic Fatty Liver Disease: A Pilot Study

**DOI:** 10.3389/fendo.2022.917304

**Published:** 2022-06-28

**Authors:** Jinying Xia, Guang Jin, Qifeng Hua, Shihan Cui, Jianhui Li

**Affiliations:** ^1^ Department of Endocrinology, Hwa Mei Hospital, University of Chinese Academy of Sciences, Ningbo, China; ^2^ Department of Ultrasound, Hwa Mei Hospital, University of Chinese Academy of Sciences, Ningbo, China; ^3^ Department of Radiology, Hwa Mei Hospital, University of Chinese Academy of Sciences, Ningbo, China

**Keywords:** diabetes mellitus, type 2, non-alcoholic fatty liver disease, liver cirrhosis, disease progression, nomograms

## Abstract

**Background:**

Correct identification of the fibrosis progression risk is a critical step in the management of patients with type 2 diabetes mellitus (T2DM) and non-alcoholic fatty liver disease (NAFLD), because liver fibrosis, especially advanced liver fibrosis, is difficult to reverse. However, the progression of liver fibrosis is typically unnoticeable, leading to many patients failing to adhere to long-term therapeutic interventions. Reliable clinical tools for the quantification of the fibrosis progression risk may have effects on following long-term therapeutic recommendations to avoid further liver injury.

**Objective:**

This study aims to develop a nomogram for quantitatively estimating the risk of fibrosis progression in T2DM patients with NAFLD during lifestyle intervention.

**Methods:**

A total of 432 medical records of T2DM patients with NAFLD were retrospectively analyzed in this study. We divided patients into the progression and no-progression groups according to whether the value of liver stiffness measurement (LSM) increased by > 2 kPa at the last visit. The independent factors associated with the fibrosis progression, which were screened by univariate and multivariate Logistic regression, constituted the nomogram to determine the likelihood of fibrosis progression in T2DM patients with NAFLD.

**Results:**

Sixty-five of the 432 individuals (15%) were found to have fibrosis progression. Changes in body mass index [odds ratio (OR) = 1.586], glycosylated hemoglobin A1c (OR = 6.636), alanine aminotransferase (OR = 1.052), and platelet counts (OR = 0.908) were independently associated with fibrosis progression (all *P* < 0.05) and functioned as components of the newly developed nomogram. It showed satisfied discrimination and calibration after 1,000 bootstrapping. The DCA indicated that the nomogram yielded clinical net benefit when the threshold probability was < 0.8.

**Conclusion:**

We developed a nomogram incorporating dynamic alterations in clinical features to estimate the risk of fibrosis progression in T2DM patients with NAFLD, which aids the patients’ compliance with long-term life interventions while allowing for prompt intervention adjustments.

## Introduction

Non-alcoholic fatty liver disease (NAFLD) usually coexists with type 2 diabetes mellitus (T2DM) due to the bidirectional association with components of the metabolic syndrome (1, 2). The prevalence of NAFLD in the general population ranges from 20% to 30%, while it is 47.3-63.7% in the T2DM population (3). The fibrosis stage is identified as a key determinant of the long-term outcome in NAFLD patients (4, 5). A well-developed meta-analysis revealed that the likelihood of liver-related and all-cause mortality increases with fibrosis stages compared with NAFLD patients without fibrosis (6). Patients with advanced fibrosis are at a higher risk for progressing to decompensated cirrhosis, portal hypertension, and even hepatic carcinoma (7). Recent studies revealed that diabetic patients with NAFLD are more likely to have a high prevalence of advanced fibrosis (8–10). A recent meta-analysis indicated that 17% of T2DM patients with NAFLD have advanced fibrosis, which was almost twice the rate of the general population (11). Although liver fibrosis may be reversible after receiving timely and appropriate intervention (12), reversal often occurs too slowly or too infrequently to avoid life-threatening complications, particularly in advanced fibrosis (13). Therefore, correct identification of patients at the risk of fibrosis progression is a critical step in the management of diabetic patients with NAFLD.

Lifestyle intervention remains the first-line therapy for diabetic patients with NAFLD, and it was shown to be associated with the improvements in all histological outcomes including liver fibrosis (14). However, it is worth noting that in a longitudinal cohort study using paired liver biopsies, 14 (27%) NAFLD patients [8 simple steatosis, 5 borderline nonalcoholic steatohepatitis (NASH), and 1 NASH at baseline] were reported to have fibrosis progression in 3 years (15). This may be due to insufficient adherence to long-term therapeutic interventions in many NAFLD patients and the fact that no symptoms are common features of NAFLD (16, 17). We believe that the lack of reliable clinical tools for the quantification of the risk of fibrosis progression is the possible explanation, leading to a decline in willpower in these patients throughout their treatment (18). Hence, there is a great demand to develop a reliable noninvasive tool to quantitatively evaluate the risk of progressive fibrosis in T2DM patients with NAFLD, which may have effects on following long-term therapeutic recommendations to at least avoid fibrosis progression.

Although liver biopsy is the gold standard of liver fibrosis diagnosis, it is invasive and unsuitable for routine monitoring of disease progression in NAFLD patients (19). Moreover, patients undergoing liver biopsies are fundamentally different from regular NAFLD patients. Given the limitations of liver biopsies, a variety of noninvasive liver fibrosis evaluations, including prediction scores and imaging approaches, have been developed to determine the degree of liver fibrosis (20, 21). Several scoring systems based on clinical and serological variables, such as body mass index (BMI), aspartate aminotransferase to platelet ratio index (APRI), NAFLD fibrosis score (NFS), and fibrosis-4 score (FIB-4), have been developed for fibrosis assessment in recent years (22, 23). In general, the performance of imaging approaches is superior to prediction scores (24). Such approaches include transient elastography (TE) and magnetic resonance elastography (MRE). Given that MRE is not suitable as a first-line approach due to its cost and complexity, TE has been regarded as an accurate and repeatable screening approach for liver fibrosis because of its excellent association with the stage of fibrosis determined by concurrent liver biopsy (25, 26). Although these noninvasive evaluations are applicable for excluding subjects without advanced fibrosis (22, 27), their performance in tracking changes in the risk of fibrosis progression over time has yet to be validated.

With this background, the present study aims to develop a nomogram-based non-invasive model for quantitatively evaluating the risk of fibrosis progression in T2DM patients with NAFLD. We selected this patient population because of their higher risks compared to patients with nondiabetic NAFLD. Timely and effective treatment adjustment in the therapeutic interventions may allow them to avoid further liver injury.

## Materials and Methods

This study was planned in accordance with the declaration of Helsinki. The institutional review board of Hwa Mei Hospital, University of Chinese Academy of Sciences approved this study (YJ-NBEY-KY-2021-081-01) and informed consents were obtained from all patients.

### Patient Selection

This retrospective study was performed for adult patients with a diagnosis of T2DM combined with NAFLD who had undergone liver stiffness measurement with TE at Hwa Mei Hospital, University of Chinese Academy of Sciences during the period from January 2017 to January 2022. All patients received recommendations for weight loss with lifestyle changes such as hypocaloric diets and physical exercise, and were taking antidiabetic and antihypertensive medications as appropriate. The diagnosis of T2DM was established according to American Diabetes Association criteria (28). As required by international associations, NAFLD is diagnosed when (a) there is evidence of hepatic steatosis indicated by imaging or histology, (b) there are no viral hepatitis, autoimmune hepatitis, or other known causes of liver disease, and (c) there are no causes for secondary hepatic fat accumulation, such as significant alcohol intake (>30g/day for males and >20g/day for females) and use of steatogenic medication (1, 2).

Liver stiffness measurement (LSM) with TE was routinely performed for all NAFLD patients in our institution at initial diagnosis and during follow-up since 2016. The inclusion criteria of this study were: 1) patients with an initial LSM value < 7.1 kPa, which was reported by Eddowes et al. (26) to rule out advanced fibrosis (NPV of 0.89), and 2) patients were followed up for more than 3 years. Those patients were excluded based on the following criteria: 1) malignancies, 2) significant alcohol intake during follow-up, 3) missing or incorrect data, and 4) lost to follow-up.

**Figure 1:**
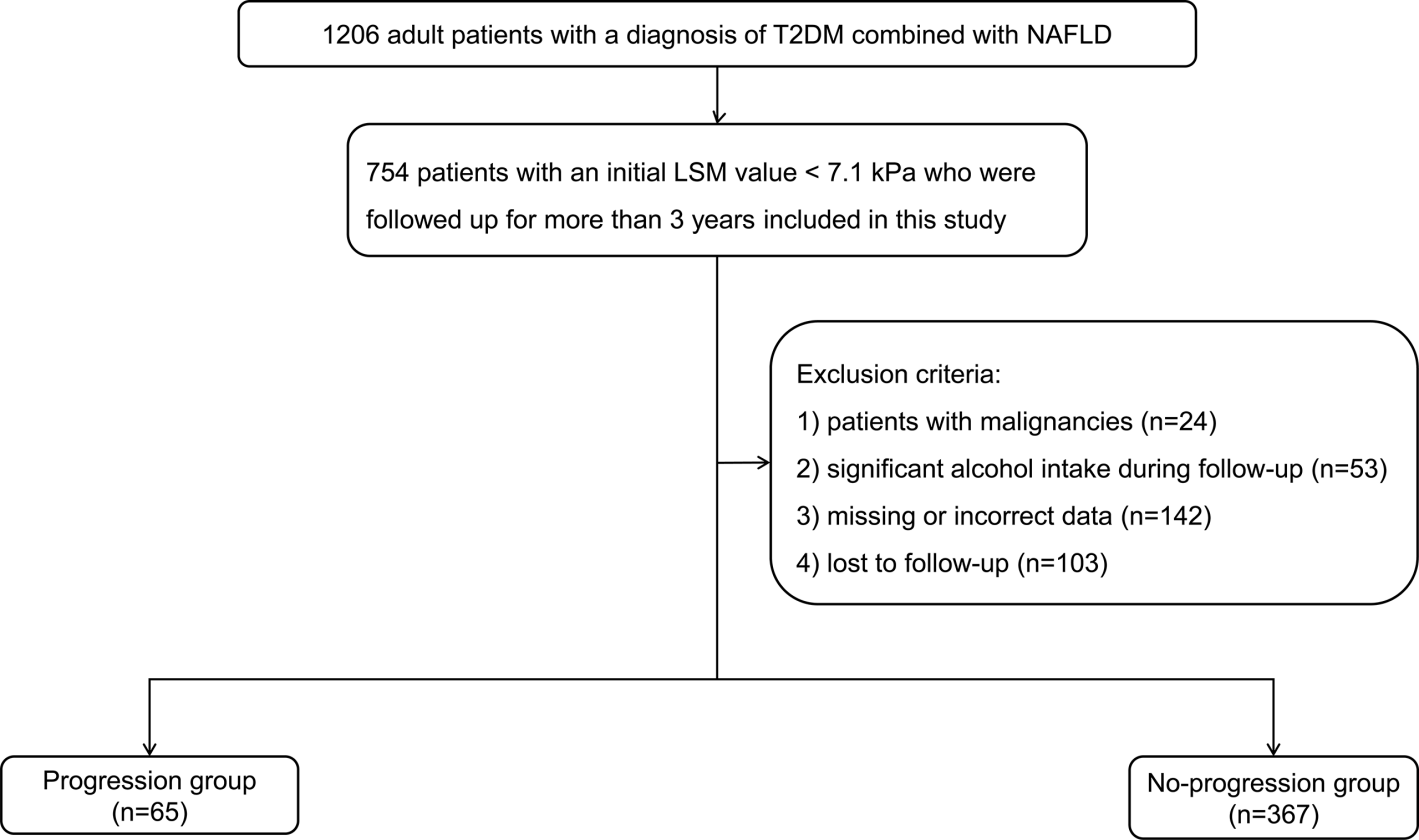
Flowchart of patient selection and grouping. T2DM, type 2 diabetes mellitus; NAFLD, non-alcoholic fatty liver disease; LSM, liver stiffness measurement.

Ultimately, 432 strictly screened medical records of T2DM patients with NAFLD were enrolled in this study. A flow diagram illustrating patient selection and grouping is shown in **Figure 1**. We divided patients into the progression and no-progression groups according to whether the LSM value increased by > 2 kPa at the last visit.

### Clinical Evaluation

All patients were interviewed and underwent anthropometric as well as laboratory measurements. Their demographic features (age and gender), medical history, as well as clinical data at baseline and last follow-up, were recorded. Laboratory data included aspartate aminotransferase (AST), alanine aminotransferase ratio (ALT), platelet counts (PLT), albumin (ALB), total bilirubin (TB), fasting blood glucose (FBG), glycosylated hemoglobin A1c (HbA1c), total cholesterol (TC), triglyceride, high density lipoprotein cholesterol (HDL-C), and low density lipoprotein cholesterol (LDL-C). Homeostasis model assessment of insulin resistance (HOMA-IR), FIB-4, and NFS were also calculated.

### LSM

LSM values were obtained using the FibroScan 502 machine (Echosens, Paris, France) with M and XL probes by two sonographers with more than 10 years of diagnostic experience in abdomen ultrasound who were blinded to each patient’s biochemical and clinical data. Briefly, all patients are required to fast for at least 3h prior to the examination. After subjects placed in a supine position, measurements were performed by scanning the right hepatic lobe through an intercostal space. The automatic selection tool of the ultrasonic system selected the appropriate probe for each patient according to a real-time assessment of the skin-to-liver capsule distance. LSM was considered reliable only when at least 10 acquisitions were successful and the interquartile range - to - median ratio was ≤ 0.3. The LSM value recorded for each patient was the median of these valid acquisitions.

### Sample Size Estimation

The sample size was calculated using an online sample size calculator provided by the University of California San Francisco (https://sample-size.net/sample-size-conf-interval-proportion/). For successful prediction modeling, at least 10 individuals with the event of interest are required per candidate variable to avoid overestimating the predictive performance (29). In this study, we estimated the sample size using an expected proportion (P) of 0.15, a total width of confidence interval (W) of 0.07, and a confidence level (CL) of 95%. The estimated sample size was at least 400 subjects, and 432 subjects were determined to be the sample size of this study.

### Nomogram Construction

The potential variables related to fibrosis progression in the progression and non-progression groups were compared and served for univariate Logistic analysis. To explore the independent factors associated with the fibrosis progression, the multivariate Logistic regression analysis only included the significant variables from the univariate analysis (*P* < 0.05). A nomogram was then constructed based on the screened variables, which allowed us to determine the likelihood of fibrosis progression in T2DM patients with NAFLD.

### Statistical Analysis

Data are expressed as mean ± standard deviation, median (25,75 percentiles), or number (percentage) as appropriate. Differences between groups were assessed using the Student t test, Mann-Whitney U test, and chi-squared test. The independently influencing factors and their odds ratios (ORs) as well as 95% confidence interval (CI) constituted the nomogram model. Internal validity and adjustment for overfitting of the nomogram were implemented with a bootstrap resampling (1,000 times) analysis. The discrimination and calibration of the model were plotted using receiver operating characteristic (ROC) curves and calibration curves, and were assessed by the area under the ROC curve (AUC) and Hosmer-Lemeshow (HL) test. A decision curve analysis (DCA) was applied to evaluate the net benefit of our model. All statistical tests were performed using SPSS software (Version 22.0), Medcalc (Version 22.0.22), and R package (Version 3.6.2).

## Results

### Clinical Characteristics at Baseline and Changes After 3-Year Follow-Up

The medical records of 432 T2DM patients with NAFLD were enrolled in this study. Sixty-five of 432 patients were considered to have fibrosis progression due to an increase in LSM value > 2 kPa at the last visit, representing an incidence of 15%. They were divided into the progression group and the others were in the no-progression group. **Table 1** summarizes the potential variables related to fibrosis progression at baseline and changes after 3-year follow-up in T2DM patients with NAFLD. Univariate Logistic regression revealed that changes in waist circumference, BMI, HbA1c, ALT, PLT, and NFS were associated with fibrosis progression (all *P* < 0.05).

**Table 1:** Potential variables related to fibrosis progression in T2DM patients with NAFLD who were followed up for more than 3 years.

Variable		Progression group (n = 65)	No-progression group (n = 367)	*P* value	Univariate Logistic regression	
					P value	*OR*
Age, years		46.4±8.3	45.4±8.9	0.366^*^	0.365	1.014
Male gender, n(%)		211 (57.5%)	34 (52.3%)	0.437^$^	0.437	0.811
Hypertension , n(%)		33 (50.8%)	176 (48.0%)	0.676^$^	0.676	1.119
Smoking , n(%)		21 (32.3%)	87 (23.7%)	0.140^$^	0.142	1.536
Waist circumference, cm	Baseline	99.8±10.9	97.1±13.9	0.134^*^	0.135	1.015
	Change	-0.1 (-2.2, 2.6)	-0.5 (-4.5, 3.4)	0.037^#^	0.030	1.035
BMI, kg/m^2^	Baseline	27.9±4.2	27.3±4.5	0.263^*^	0.262	1.034
	Change	1.0 (-0.5, 2.4)	-0.3 (-1.2, 0.5)	<0.001^#^	<0.001	1.871
FBG, mmol/L	Baseline	7.2 (5.6, 9.2)	6.5 (4.9, 7.8)	0.108^#^	0.083	1.116
	Change	-0.8 (-2.3, 0.5)	-1.0 (-2.3, 0.4)	0.674^#^	0.673	1.029
HbA1c, %	Baseline	7.3 (5.8, 9.5)	7.3 (6.2, 8.5)	0.529^#^	0.175	1.099
	Change	-0.13 (-0.30, 0.00)	-0.37 (-0.63, -0.15)	<0.001^#^	<0.001	10.865
TC, mmol/L	Baseline	4.24±1.13	4.52±1.25	0.062^*^	0.063	0.793
	Change	-0.37±0.73	-0.51±0.83	0.112^*^	0.084	1.544
HDL-C, mmol/L	Baseline	1.16±0.51	1.09±0.40	0.179^*^	0.179	1.545
	Change	-0.06±0.08	-0.05±0.07	0.500^*^	0.499	0.259
LDL-C, mmol/L	Baseline	2.82±0.62	2.71±0.82	0.186^*^	0.186	1.252
	Change	0.05±0.11	0.03±0.10	0.101^*^	0.105	35.995
Triglycerides, mmol/L	Baseline	2.20 (1.48, 2.83)	2.03 (1.38, 2.79)	0.852^#^	0.120	0.846
	Change	0.01 (-0.11, 0.15)	0.05 (-0.18, 0.12)	0.162^#^	0.132	149.294
ALT, IU/L	Baseline	37 (24, 70)	40 (29, 55)	0.678^#^	0.103	1.014
	Change	-2 (-9, 6)	-12 (-23, 0)	<0.001^#^	<0.001	1.04
AST, IU/L	Baseline	23 (16, 31)	26 (18, 43)	0.083^#^	0.120	0.977
	Change	-5 (-10, 2)	-6 (-10, 4)	0.071^#^	0.072	1.047
ALB, IU/L	Baseline	41.7±3.2	41.8±4.3	0.926^*^	0.926	0.997
	Change	-3.3±4.9	-2.0±5.3	0.055^*^	0.056	0.951
PLT, ×10^9^/L	Baseline	234±50	245±47	0.092^*^	0.093	0.995
	Change	-7.9±10.8	5.2±12.2	<0.001^*^	<0.001	0.908
HOMA-IR	Baseline	2.8 (2.0, 3.3)	2.5 (1.8, 3.1)	0.082^#^	0.133	4.162
	Change	0.0 (-0.2, 0.2)	-0.2 (-0.6, 0.1)	0.101^#^	0.070	1.616
FIB-4	Baseline	1.11±0.46	1.16±0.48	0.426^*^	0.425	0.797
	Change	-0.14±0.31	-0.17±0.28	0.463^*^	0.462	1.379
NFS	Baseline	-1.12±0.67	-1.28±0.59	0.069^*^	0.057	1.561
Change	-0.10 (-0.53, 0.32)	-0.29 (-0.74, 0.20)	0.039^#^	0.042	1.494

^*^for independent sample t-test, ^$^for chi-square test, and ^#^for Mann-Whitney U test. T2DM, type 2 diabetes mellitus; NAFLD, non-alcoholic fatty liver disease; BMI, body mass index; FBG, fasting blood glucose; HbA1c, glycosylated hemoglobin A1c; TC, total cholesterol; HDL-C, high density lipoprotein cholesterol; LDL-C, low density lipoprotein cholesterol; ALT, alanine aminotransferase ratio; AST, aspartate aminotransferase; ALB, albumin; PLT, platelet counts; HOMA-IR, homeostasis model assessment of insulin resistance; FIB-4, fibrosis-4 score; NFS, NAFLD fibrosis score.

### Factors Associated With Fibrosis Progression

**Figure 2:**
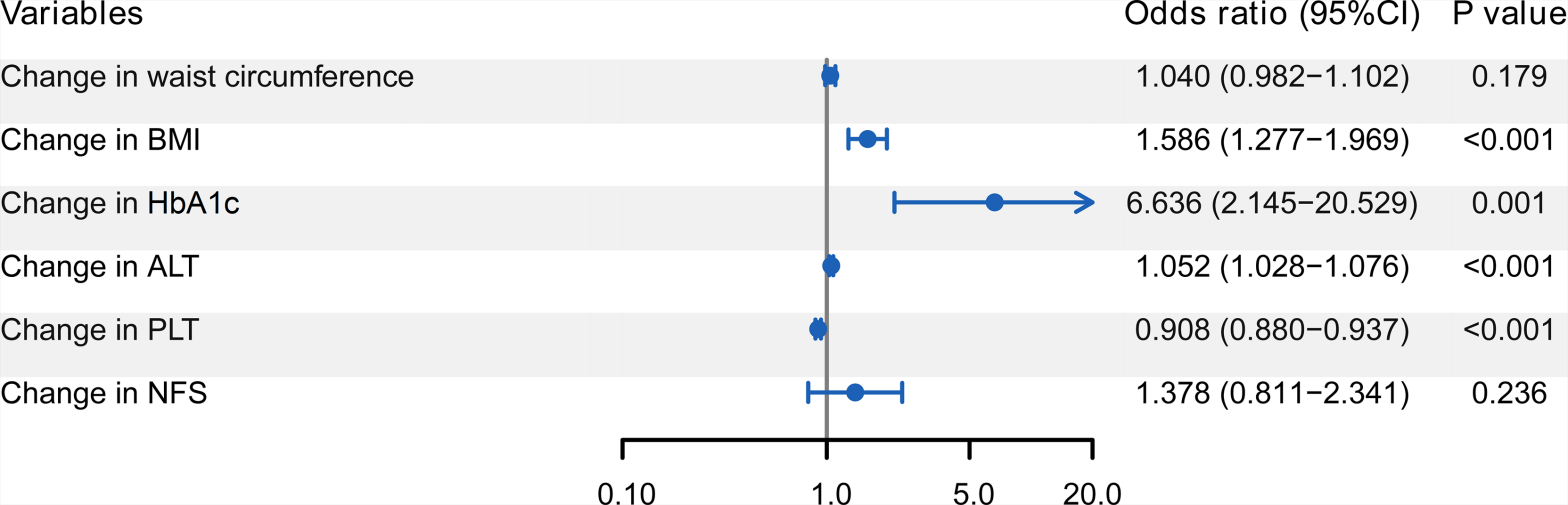
Forest plot of multivariate Logistic regression analysis for the associations between independent factors and fibrosis progression. Changes in BMI, HbA1c, ALT, and PLT are the independent factors associated with the fibrosis progression. BMI, body mass index; HbA1c, glycosylated hemoglobin A1c; ALT, alanine aminotransferase ratio; PLT, platelet counts; NFS, NAFLD fibrosis score.

Associations between each potential factor and fibrosis progression in T2DM patients with NAFLD are shown in **Figure 2**. By multivariate Logistic analysis, changes in BMI (OR = 1.586), HbA1c (OR = 6.636), ALT (OR = 1.052), and PLT (OR = 0.908) were independently associated with the fibrosis progression (all *P* < 0.05). Changes in waist circumference and NFS were uncorrelated with the fibrosis progression (both *P* > 0.05).

### Nomogram Construction

**Figure 3:**
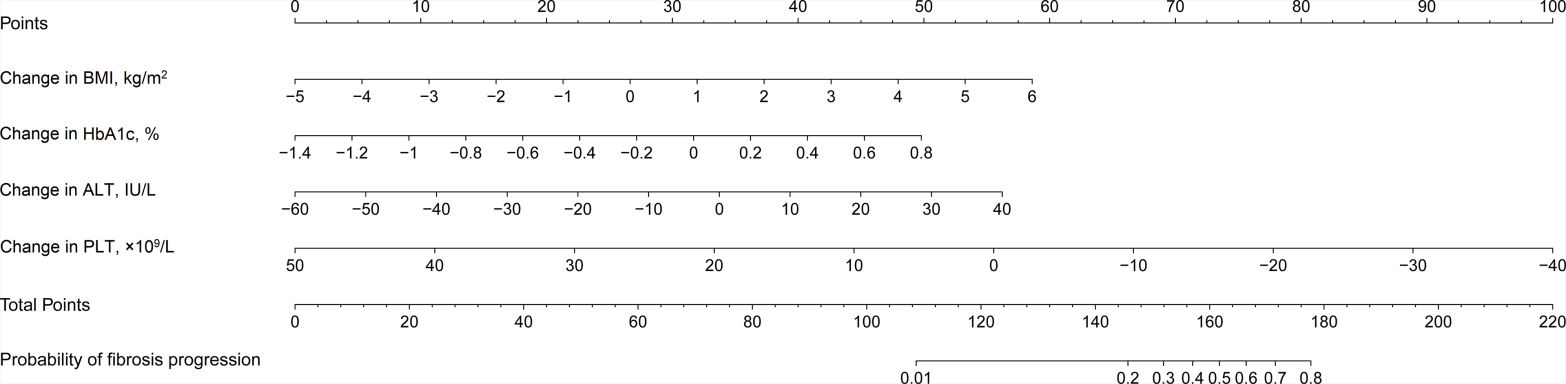
Nomogram estimating the likelihood of fibrosis progression in T2DM patients with NAFLD. T2DM, type 2 diabetes mellitus; NAFLD, non-alcoholic fatty liver disease; BMI, body mass index; HbA1c, glycosylated hemoglobin A1c; ALT, alanine aminotransferase ratio; PLT, platelet counts.

A nomogram was constructed using the results of the multivariate Logistic analysis to estimate the likelihood of fibrosis progression in T2DM patients with NAFLD (**Figure 3**). Clinical scores were assigned to the 4 independent factors and the estimated risk of progression was calculated by summing the scores of each factor, with the weight equal to the OR value. The final score ranged from a minimum of zero points to a maximum of 220 points. For example, a 55-year-old man with T2DM combined with NAFLD received a recommendation for weight loss with lifestyle changes. After 3 years, his changes in BMI, HbA1c, ALT, and PLT were 1kg/m^2^, 0.2%, 0U/L, and -10*10^9^/L, respectively. The total score was about 170, indicating that his risk of fibrosis progression was about 65%.

### Model Validation

**Figure 4:**
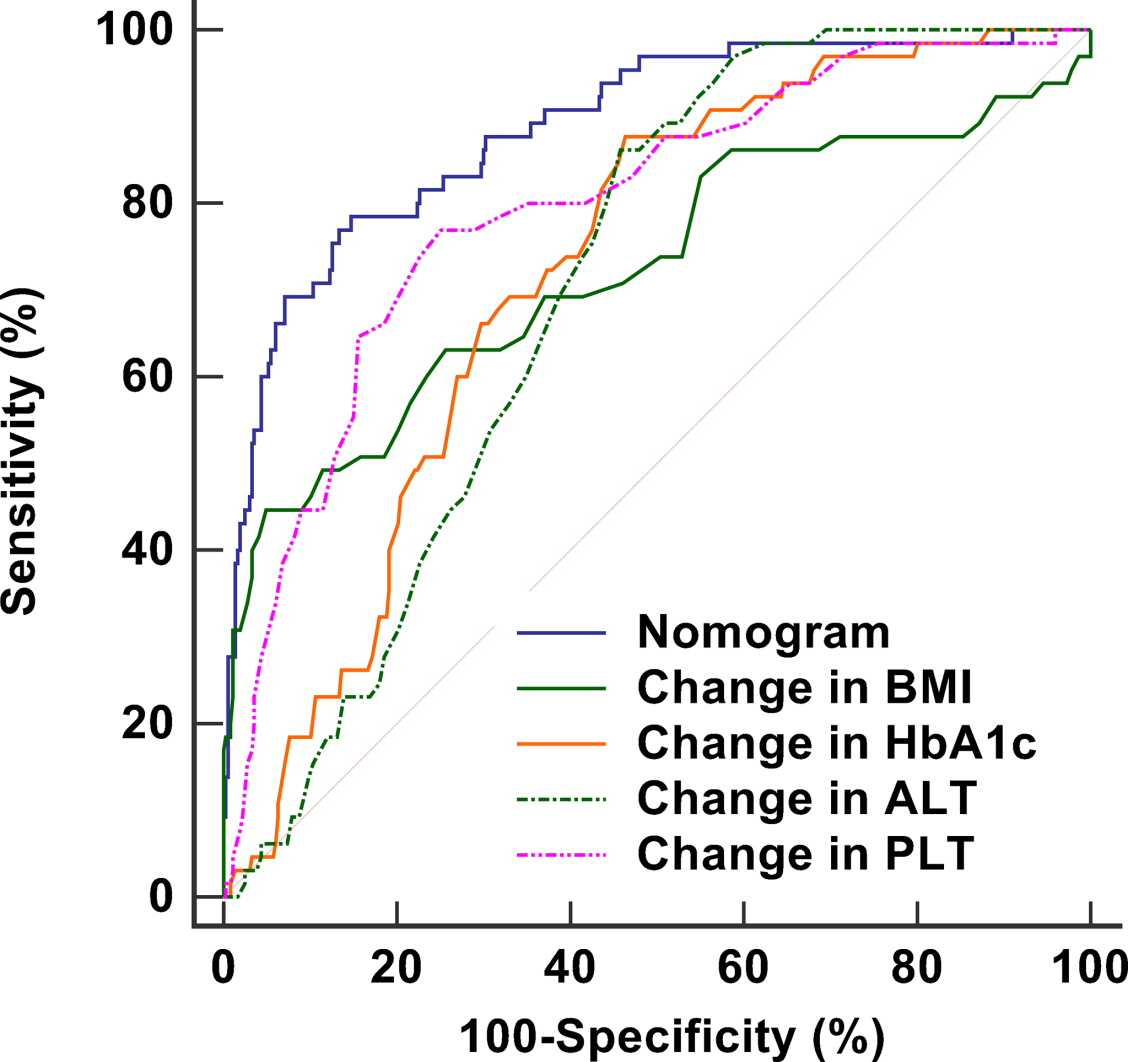
ROC curves including nomogram and its components to assess the predictive accuracy of fibrosis progression. The AUC of the nomogram is 0.887, which is higher than the AUC values for changes in BMI (0.723), HbA1c (0.721), ALT (0.703), and PLT (0.797), demonstrating satisfied discrimination. ROC, receiver operating characteristic; AUC, area under the curve; BMI, body mass index; HbA1c, glycosylated hemoglobin A1c; ALT, alanine aminotransferase ratio; PLT, platelet counts.

**Figure 5:**
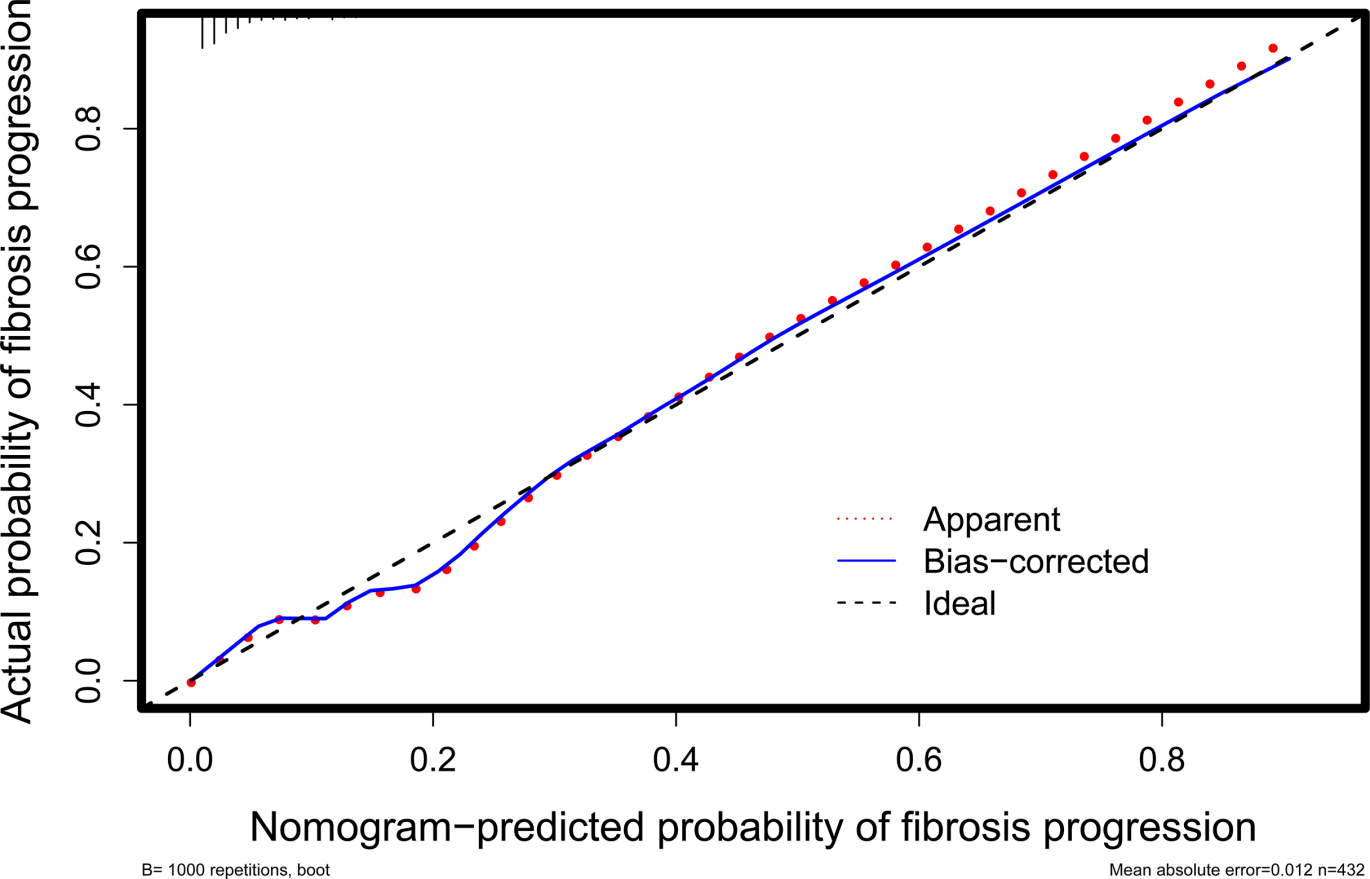
Calibration curve for evaluating the agreement between the nomogram-predicted probability and the actual probability. It shows that they are well matched, indicating good calibration of the nomogram.

The stability of the model was verified after 1,000 bootstrapping and the overfitting-corrected AUC was 0.887 (95% CI: 0.710 - 0.814) with the sensitivity and specifificity of 46.2% and 96.7%, respectively. It was higher than the AUC values for changes in BMI (0.723), HbA1c (0.721), ALT (0.703), and PLT (0.797), demonstrating satisfied discrimination (**Figure 4**). The calibration of the model including these four variables was good because of no significant difference between the predicted and actual likelihood of fibrosis progression (*χ^2^* = 12.099, *P* = 0.147) (**Figure 5** **)**.

### DCA for Clinical Utility of the Nomogram

**Figure 6:**
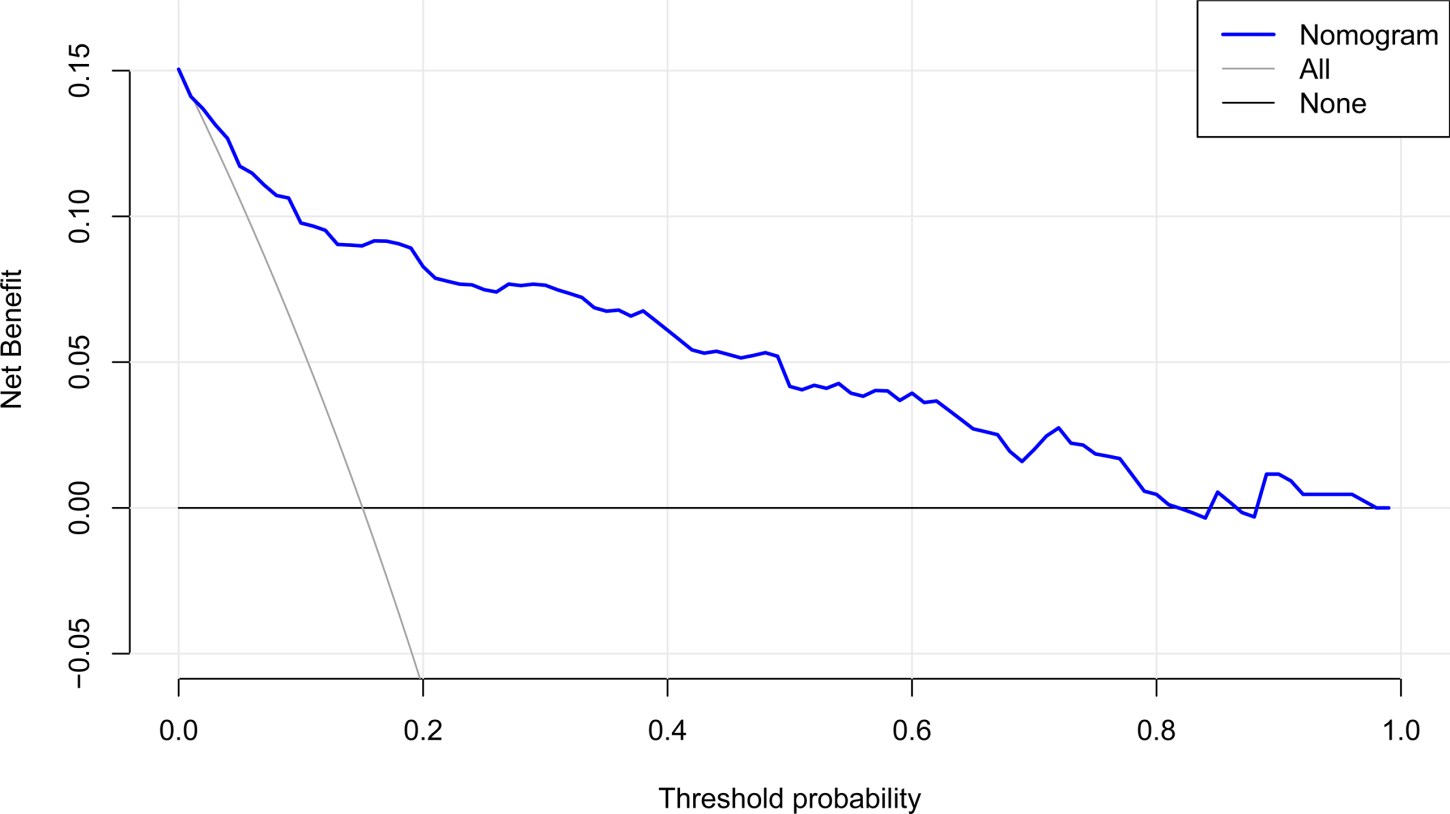
DCA of the nomogram. It indicates that the nomogram yields clinical net benefit when the threshold probability is < 0.8. DCA, decision curve analysis.

The DCA curve utilized to evaluate the clinical utility of the nomogram is plotted in **Figure 6**. The DCA revealed that, when the threshold probability was less than 0.8, a net benefit was obtained by employing the nomogram.

## Discussion

Despite the development of various non-invasive approaches for identifying or ruling out advanced fibrosis, there is still a scarcity of tools tracking changes in fibrosis progression over time. Because fibrosis progression may also occur in common NAFLD patients, it is necessary to monitor the disease progression and liver injury frequently. Therefore, it is critical to not only detect advanced fibrosis but also identify patients with progressive fibrosis during follow-up. In the present study, we developed and validated a nomogram to identify T2DM patients with NAFLD at risk of fibrosis progression. Our study revealed that changes in BMI, HbA1c, ALT, and PLT were independent factors for fibrosis progression. The greatest strength of this study is that the components of our nomogram were the dynamic variations with respect to baseline characteristics. Individualized assessment of the fibrosis progression risk aids the patients’ compliance with long-term life interventions while also allowing for timely and effective intervention adjustments for them.

According to our findings, simple hepatic steatosis in T2DM individuals was not always quiescent. Despite the fact that we excluded patients with advanced fibrosis based on an initial LSM value of 7.1 kPa (26), 15% of patients experienced fibrosis progression after three years. This proportion was significantly higher than the increase of histological fibrosis stage in common NAFLD patients. A recent meta-analysis revealed that it took an average of 14.3 years to progress by one stage of fibrosis in patients with NAFLD (30). In this study, we defined a 2 kPa increase in the LSM value as fibrosis progression, although it was unclear whether it was associated with histological progression of liver fibrosis. This is because for patients with an initial LSM value < 7.1 kPa, an increase of 2 kPa is equivalent to a 30% increase, implying that it is related to an increase in liver stiffness rather than a measurement error. Moreover, according to the analysis about the diagnostic accuracy of LSM by Eddowes et al. (26), an increase of 2 kPa can be regarded as one stage of liver fibrosis in patients. A recent study on fibrosis progression also reported that 85.7% of individuals with fibrosis progression had an LSM increase of ≥2 kPa (31).

In this investigation, changes in BMI, HbA1c, ALT, and PLT were revealed to be the independent factors of fibrosis progression in T2DM patients with NAFLD. They are all recognized factors and appear in multiple scoring systems. Obesity and diabetes, particularly poor BMI and HbA1c control, exacerbate the natural course of NAFLD and are linked to advanced liver fibrosis (8, 32). Although the effects of lifestyle changes were not systematically analyzed, our findings support the idea that reductions in BMI and HbA1c are beneficial in NAFLD patients. Recent research has found a close relationship between BMI and HbA1c improvement and histological regression or static fibrosis stage (33–35). Our study showed that ALT and PLT were consistently correlated with the fibrosis progression, regardless of BMI and HbA1c, implying a direct relationship between ALT and platelet levels and fibrosis progression. Based on our findings, changes in BMI, HbA1c, ALT, and PLT are useful in assessing the antifibrotic efficacy into the context of lifestyle interventions. The greatest challenge is how to sustain a healthy lifestyle and its positive impacts on long-term therapeutic procedures (36).

The commonly used scores had limited accuracy in detecting changes in fibrosis stage with time (15). Several studies have recently been conducted to predict fibrosis change in NAFLD patients (15, 35). However, they are not applicable to most NAFLD patients because paired liver biopsies were utilized to evaluate their disease progression. To our knowledge, this study is the first to apply a nomogram in regular patients with T2DM and NAFLD to estimate the risk of fibrosis progression during long-term life interventions. Our model was developed to enhance individuals’ adherence and willpower to maintain a healthy lifestyle. This nomogram showed satisfied discrimination and calibration performance, as well as a significant net benefit in detecting individuals at risk of fibrosis progression. As the example described in the result section, he had a 65% risk of fibrosis progression, implying that active intervention to prevent further progression might be required. In addition, some medications, such as cenicriviroc and obeticholic acid, may help to reverse liver fibrosis (37, 38).

There are several limitations in this study. First, no liver biopsy was conducted because performing biopsies on every NAFLD patient is unethical and impractical. TE is now available in America, Europe, and Asia with a satisfactory diagnosis compared to liver biopsy (23, 39). Second, This study was retrospective in design and the sample size was limited due to the strict inclusion and exclusion criteria, which was inevitably susceptible for selection bias. Finally, there was no external validation for our nomogram, despite our confidence that it will perform well in identifying patients at risk of fibrosis progression. A multicenter investigation should be implemented in the future to remedy the absence of a validation study.

## Conclusion

Our findings revealed that changes in BMI, HbA1c, ALT, and PLT after 3 years of lifestyle intervention were associated with fibrosis progression in T2DM patients with NAFLD. We developed a noninvasive nomogram including these variables to quantitatively estimate the risk of fibrosis progression. It allows patients to adjust medical interventions early and improves adherence to lifestyle interventions before progression to advanced fibrosis.

## Data Availability Statement

The raw data supporting the conclusions of this article will be made available by the authors, without undue reservation.

## Ethics Statement

The studies involving human participants were reviewed and approved by Ethical approval for the study was obtained from the ethics committee of Hwa Mei Hospital, University of Chinese Academy of Sciences YJ-NBEY-KY-2021-081-01. The patients/participants provided their written informed consent to participate in this study.

## Author Contributions

Study design: JX, GJ, and JL. Data collection and analysis: JX, GJ, QH, and SC. Supervision: JX and JL. Statistics: JX, GJ, QH, and SC. Manuscript writing: JX, GJ, QH, SC, and JL. Manuscript revision: JX and JL. Approval of the manuscript: all authors.

## Funding

Medical Scientific Research Foundation of Zhejiang Province, China (2020KY854), Medical Scientific Research Foundation of Zhejiang Province, China (2018KY696), Ningbo Public Service Technology Foundation, China (202002N3184), and Research Foundation of Hwa Mei Hospital, University of Chinese Academy of Sciences, China (2018HMKY27).

## Conflict of Interest

The authors declare that the research was conducted in the absence of any commercial or financial relationships that could be construed as a potential conflict of interest.

## Publisher’s Note

All claims expressed in this article are solely those of the authors and do not necessarily represent those of their affiliated organizations, or those of the publisher, the editors and the reviewers. Any product that may be evaluated in this article, or claim that may be made by its manufacturer, is not guaranteed or endorsed by the publisher.
